# To beta block or not to beta block; that is the question

**DOI:** 10.1186/s13054-015-1059-6

**Published:** 2015-09-24

**Authors:** Can Ince

**Affiliations:** Department of Intensive Care, Erasmus MC, University Medical Center, s-Gravendijkwal 230, 3015 CE Rotterdam, The Netherlands

## Abstract

The fast-acting β-1 blocker esmolol has been the center of attention since the landmark article by Morrelli and colleagues suggesting that, in patients with sepsis, reducing heart rate by administering esmolol can result in a survival benefit. However, the use of esmolol for the treatment of sepsis and the underlying mechanism responsible for this benefit remain controversial. This commentary discusses the study by Jacquet-Lagrèze and colleagues, who in a pig model of sepsis tested the hypothesis that administration of esmolol to reduce heart rate may correct sepsis-induced sublingual and gut microcirculatory alterations which are known to be associated with adverse outcome.

Sepsis is one of the most complex syndromes to manage in clinical medicine today. This is because its pathophysiology is incompletely understood. It is characterized by an inflammatory storm leading to cardiovascular compromise resulting in cellular dysfunction and ultimately to organ failure. Its pathogenesis changes rapidly in time, and so monitoring its progress is challenging because conventional monitoring devices are able to measure only systemic hemodynamic variables whereas the pathogenesis of sepsis resides at the microcirculation and the parenchymal cells. Given this background it is understandable that the search for effective therapeutic strategies remains wanting since it is difficult to assess whether the physiological concept on which a therapy is based—which ultimately must be related to improved parenchymal perfusion, oxygenation, or function or a combination of these—is indeed being affected. That is why experimental investigations in animal models, which provide a deeper insight into underlying mechanisms, are helpful. If novel monitoring techniques which can be translationally applied to patients are also used, such studies are especially relevant for the introduction of new treatment modalities.

In such a study, Jacquet-Lagrèze and colleagues investigated the possible beneficial effect of the fast-acting β-1 blocker, esmolol, in improving sublingual and gut microcirculation in a porcine model of sepsis [1]. The study is opportune because, even though there is a theoretical benefit for administering a β-blocker to reduce heart rate (HR) and control the adverse effects of an adrenergic storm associated with sepsis, there is uncertainty about which precise mechanism affected by β-blockers causes a potential therapeutic benefit. Fuelled by the improved survival study in patients with sepsis by Morelli and colleagues [2], various mechanisms of action, including hemodynamic, inflammatory, metabolic, and coagulation effects, have been proposed [3]. The most obvious advantage for the use of a β-blocker, however, remains the expected preservation or improvement of stroke volume because of a longer diastolic filling time associated with the reduction in HR. However, even though almost all studies show an HR reduction following esmolol administration, conflicting effects on cardiac hemodynamics have been reported. Morelli and colleagues, in their original study, reported that the expected increase in stroke volume in patients with esmolol-treated sepsis was associated with a reduction in HR [2]. In a subsequent study, however, in which they reported an improved sublingual microcirculation in response to esmolol, they found no such increase in stroke volume [4]. In a porcine model of hypodynamic sepsis, Aboab and colleagues found that esmolol causes a stroke volume increase associated with a reduced HR [5]. In the present study, however, also in a porcine model, Jacquet-Lagrèze and colleagues found that the reduction in HR was not accompanied by a rise in stroke volume. In short, there seems to be much confusion about what esmolol actually does to septic cardiac hemodynamics. Nevertheless, it cannot be denied that tachycardia in critically ill patients has a bad prognosis and treating it (for example, by a β-blocker) remains an attractive option. Indeed, in a multi-center international observational trial in 530 mixed intensive care patients, we showed that tachycardia was the single most sensitive parameter for predicting adverse outcome [6]. If in addition to tachycardia there were signs of microcirculatory alterations as measured by the same handheld microscopes used in the present study, prognosis for adverse outcome increased by 80 %.

In their study, Jacquet-Lagrèze and colleagues set out to achieve a hyperdynamic sepsis model by infusing live bacteria to target an increase in pulmonary arterial pressures [1]. Though effective in increasing pulmonary arterial pressure, this strategy did not significantly alter any systemic or metabolic parameters, and their model could better be described as a normotensive model of sepsis. Of specific interest and relevance of their septic model, however, was the finding that, despite normal systemic and cardiac hemodynamics, severe microcirculatory alterations were observed sublingually and in the gut, a condition similarly reported in several clinical studies in sepsis and shown to be associated with increased morbidity and mortality [7, 8]. The response to esmolol in their study, however, had marginal effect on the sublingual and gut microcirculation, which remained depressed. This finding in itself is important because it suggests that, even though systemic hemodynamic variables may be within the boundaries of normality, sepsis-induced microcirculatory alterations are not corrected by esmolol despite a reduction in HR. Nevertheless, the authors somewhat surprisingly conclude that “esmolol provided a maintenance of microcirculation, despite its negative aspects on macrocirculation” [1]. Instead, it may be more correct to conclude that esmolol, despite providing a reduction in HR, showed no benefit in their normotensive model of sepsis with microcirculatory alterations. There remains the explanation, though statistically not significant, regarding the trend to improved gut microcirculation. This could have been caused by a redistribution of blood flow in favor of the gut. Measurement of arterial mesenteric blood flow would have been helpful in this context. This trend to improved gut microcirculation in the group statistics could also be explained by the increased use of milrinone in the esmolol group. This is significant because other inodilators such as levosimendan have been described in clinical and animal studies at improving microcirculatory perfusion and tissue oxygenation [9, 10]. For a true evaluation of their results, it would have been better to exclude the experiment in which milrinone was administered. In addition, though not investigated, the slight increase in gut microcirculation could be attributed to the anti-inflammatory effect of esmolol in terms of reported reductions of tumor necrosis factor-alpha and nuclear factor-kappa-beta in animal models of sepsis [11, 12]. Despite these considerations, the lack of improvement of sublingual microcirculation in their study remains worrying and does not bode well for these septic pigs since a sustained depressed sublingual microcirculation is a well-known indicator of adverse outcome. But this does leave unanswered the question of whether β-1 blockers are indeed of benefit in the treatment of hemodynamic alterations associated with sepsis. That is still the question.

## Abbreviation

HR: Heart rate
